# Ignimbrites Related to Neogene Volcanism in the Southeast of the Iberian Peninsula: An Experimental Study to Establish Their Pozzolanic Character

**DOI:** 10.3390/ma16041546

**Published:** 2023-02-13

**Authors:** Domingo A. Martín, Jorge L. Costafreda, Leticia Presa, Elena Crespo, José Luis Parra, Beatriz Astudillo, Miguel Ángel Sanjuán

**Affiliations:** 1 Escuela Técnica Superior de Ingenieros de Minas y Energía, Universidad Politécnica de Madrid, C/Ríos Rosas, 21, 28003 Madrid, Spain; 2 Laboratorio Oficial para Ensayos de Materiales de Construcción (LOEMCO), C/Eric Kandell, 1, 28906 Getafe, Spain; 3 Dpto, Mineralogia y Petrologia, Fac. CC. Geologicas, Universidad Complutense de Madrid, C. de José Antonio Novais, 12, 28040 Madrid, Spain; 4 Department of Science and Technology of Building Materials, Civil Engineering School, Technical University of Madrid, 28040 Madrid, Spain

**Keywords:** ignimbrites, pozzolans, pozzolanicity, cements, mortars, mechanical strength, sustainable development goals, sustainability, sustainable building materials, eco-cements

## Abstract

The speed at which climate change is happening is leading to a demand for new pozzolanic materials that improve the quality of cements and, at the same time, limit the emission of greenhouse gases into the atmosphere. The main objective of this work is the detailed characterization of an ignimbrite sample (IGNS) to demonstrate its effectiveness as a natural pozzolan. To meet this objective, a series of tests were carried out. In the first stage, mineral and chemical analyses were performed, such as petrographic analysis by thin section (TSP), X-ray diffraction (XRD), oriented aggregate (OA), scanning electron microscopy (SEM) and X-ray fluorescence (XRF). In the second stage, the following technical tests were carried out: chemical quality analysis (QCA), pozzolanicity test (PT) and mechanical compressive strength (MS) at 7, 28 and 90 days, using mortar specimens with ignimbrite/cement formulation (IGNS/PC): 10, 25 and 40% to establish the pozzolanic nature of the ignimbrite. The results of the mineral and chemical analyses showed that the sample has a complex mineralogical constitution, consisting of biotite mica, potassium feldspar, plagioclase, smectite (montmorillonite), quartz, volcanic glass, iron, titanium and manganese oxides, chlorite and chlorapatite. On the other hand, the technological tests revealed the pozzolanic nature of the sample, as well as visible increases in the mechanical compressive strengths in the three proportions, the most effective being IGNS/PC:10% and IGNS/PC:25% at 7, 28 and 90 days of setting. The results obtained could be applied in the formulation of new pozzolanic cements with ignimbrite as a natural pozzolanic aggregate.

## 1. Introduction

Ignimbrites have been widely studied and applied in many fields as building materials, as natural aggregates in cements and concrete, and as pozzolans in mortar mixtures. Testimony of this is given by Hattatoglu and Bakis [1], who have incorporated ignimbrite powder in the dosage of concretes used in road construction, achieving mechanical compressive strengths of up to 124.99 MPa and flexure of 9.18 MPa. Likewise, Conde and Milestone [2] have managed to demonstrate the effectiveness of ignimbrite in the preservation of mortars subjected to degradation processes in geothermal environments enriched in CO_2_ and attribute this phenomenon to the presence of large pores in the ignimbrites making the hydraulic reaction of cement with this rock more effective over time. The same authors [3] have extended their research to the interaction that occurs between cement and ignimbrite in geothermal wells, emphasizing the effect caused by the addition of silica in cement and the impact caused by brine enriched in CO_2_, concluding that the presence of CO_2_ significantly influences the degree of interactivity of the cement–ignimbrite interface. Sepúlveda et al. [4] detail the effect of dynamic loading on the shear resistance of ignimbrites during seismic processes and the consequence it has on structural changes in soil, such as landslides of large land masses. Laçin [5] has carried out petrographic and chemical studies on ignimbrites and gypsum contained in mortars to understand and reconstruct the construction skills and techniques used in the Mid Chalcolithic. Rispoli et al. [6] have studied the fragments of ignimbrites included in the ancient mortars of an archaeological complex located in the distal surroundings of the city of Pompeii, using archaeometric techniques, concluding that the conservation of these mortars is largely due to the pozzolanic nature of the ignimbrites. Bina’s studies [7] seek to monitor the nature of the alkali–silica reaction between aggregates composed of ignimbrite and cement, showing that this rock has an innocuous behavior in this reaction and a positive effect on the formation of stable secondary compounds. Işik et al. [8] have successfully established the use of ignimbrite as a building material in the form of highly reactive fine aggregates in mixtures with concrete. Along the same lines, Izzo et al. [9] have demonstrated the effectiveness of ignimbrite as an efficient aggregate in mortars used to restore historical monuments. Columbu and Garau [10] have researched this field using mineralogical, petrographic, and chemical studies of samples of ignimbrite used in antiquity in the construction of the Roma Nora Theatre. Novembre et al. [11] emphasize the importance of ignimbrite as a high-quality cementitious material. This quality is attributed to the presence of zeolitic secondary mineral phases, which infer a strong pozzolanic nature to ignimbrite and a high hydraulic reactivity to the cement–pozzolan interface within the mortar. In the study area, the investigations carried out by Costafreda [12], Pelayo [13] and Pelayo et al. [14] mention the presence of ignimbrites in a geological enclave known as Morrón de Mateo, where these rocks are found to have a with a strong genetic and spatial link with powerful bentonite formations, currently in exploitation.

The purpose of this research is the detailed study of the mineralogical, petrographical, chemical and mechanical characteristics of ignimbrites lying in the southeast of the Iberian Peninsula in order to establish their character as pozzolans and their capacity to partially substitute for Portland cement. On the other hand, this research aims to diversify the knowledge and use of most of the volcanic materials related to Neogene volcanism in Spain.

The present study could be considered novel in the sense that it demonstrates that ignimbrites also possess evident pozzolanic properties, thus constituting an additional contribution to the knowledge of new sustainable natural materials for the preservation of the environment. On the other hand, this research could be an effective tool in the methodological selection of ignimbrites as a natural substitute for Portland cement, thus contributing to the reduction of CO_2_ emissions into the atmosphere.

## 2. Materials and Methods

### 2.1. Materials

**Figure 1:**
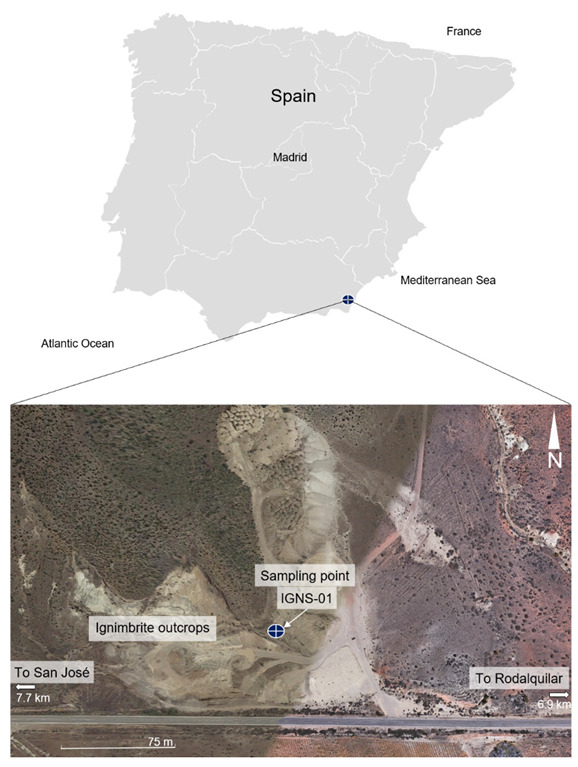
Location of the study area indicating the sampling point. Based on information from the Google Earth platform [15].

The sample studied comes from the volcanic region of the southeast of the Iberian Peninsula, specifically at a place near Morrón de Mateo, next to the road that connects San José with Rodalquilar (Figure 1). The sample is in a state of visible alteration, with a strong tendency for disintegration. To carry out this research, a representative amount of sample, equivalent to 50 kg, was taken directly from an outcrop, using the channel sampling method.

A Portland cement (PC) of Type I and Resistance class CEM I 42.5 R was used in this research. All the quality parameters and procedures for using this cement are outlined in the Standard UNE EN 197-1:2011 [16]. A standardized sand (SS), with the official designation CEN-NORMSAND DIN EN 196-1, was used in the mechanical strength tests as a fine aggregate, as indicated in the Standard UNE EN 196-1:2016 [17]. The requirements to be met by CEN standard sand are defined in the standard mentioned above as follows: high quartz content, standardized particle size distribution, amount present in the mortar mixture (1355 ± 5 g) and humidity below 0.2%.

### 2.2. Methods

#### 2.2.1. Characterization Analysis of Mineral and Chemical Phases Using TSP, XRD, OA, SEM and XRF

An analysis made by thin section petrography (TSP) was carried out on the ignimbrite sample to establish the presence of petrogenic mineral phases, as well as the main types of variations. The presence of variations is decisive in establishing the stability and quality of ignimbrite as a material capable of replacing cement. In this research, a petrographic microscope with a plane-polarized transmitted light was used as well as a polarized-crossed light.

Powder XRD on a Bruker D8 Advance diffractometer with a Cu anticathode from the Universidad Complutense de Madrid (Laboratory of Geological Techniques, CAI, Univer-sidad Complutense de Madrid, Madrid, Spain) was used to identify the mineral phases. Diffracto-grams were continuously recorded at 2° angles, from 2° to 68°, with one second per step and 0.02 stepping intervals. XPowder software (Madrid, Spain) was utilized for the semiquantitative analysis, which was carried out by the Chung method using XPowder software, which is based on finding the reference intensity ratios (RIR) of the existing phases. This makes it possible to normalize the intensity calculations by assuming that the sum of all the phases in the sample is 100 percent.

Samples were prepared for the oriented aggregates XRD method (OA) by dispersing an additional fraction of the original sample (0.1–0.5 g) in distilled water for further anal-ysis of the clay minerals. The fraction smaller than 0.5 m was then separated, extracted, and pipetted into three glass slides for three different preparations: (1) airdried oriented aggregates (air-dried, AD); (2) ethylene glycol–treated oriented aggregates (EG) for 24 h; and (3) thermally treated oriented aggregates (TT) for 90 min.

A Philipps Analytical PW 1752 Cu K radiation power diffractometer (graphite monochromator radiation K1 = 1.5406) was used to identify the mineral phases. Diffractograms were continuously recorded at two angles ranging from 2° to 35°, with 0.8 s per step and 0.02 stepping intervals.

According to Moore and Reynolds (1989) and Srödon (1984), the semiquantitative analysis was carried out with the help of the XPowder software. The reflective power ap-plied over the measured areas of each mineral main reflection peak serves as the basis for this semiquantitative technique, which assumes that the total number of clay minerals in the sample is 100 percent.

A Hitachi S-570 scanning electron microscope from the Centralized Laboratory of the Escuela Superior de Ingenieros de Minas y Energía of the Universidad Politécnica de Ma-drid (Madrid, Spain) was used to perform the morphological characterization of the samples. A Polaron BIORAD, a power supply for evaporation, a Kevex-1728 analyzer, and a Polaron SEM coating system are included in the equipment. The apparatus achieves an amplification of 200,103 and a resolution of 3.5 nm. Winshell and Printerface programs were used to take microphotographs and manage the data gathered from the analysis of the sample.

The samples had their diameters reduced to between 0.2 and 0.5 cm. They were ini-tially pulverized before being spread out on graphitized graphite tape that was adhered to the sample holder’s surface. The Polaron SEM Coating System was then used to apply a layer of graphite to cover the samples in a vacuum.

Using a Bruker S2RANGER spectrometer (Madrid, Spain) with a Pd tube, X-ray fluorescence was used to analyze the ignimbrite sample. The rock sample was ground up, homogenized, and pressed into a pellet using wax in a ratio of 1:10 by weight as a binder.

#### 2.2.2. Technical Characterization Analysis with QCA, PT, and MS

A chemical quality analysis (QCA) was used to identify the primary compounds of the natural ignimbrite sample (IGNS-N) analyzed for this study, as well as to identify it as a natural pozzolan and investigate its potential application as a natural aggregate in pozzolanic cement. The guidelines outlined in Standard UNE EN 196-2:2014 [18] were followed during the testing process. The following were the main compounds found: total SiO_2_ (TS), reactive SiO_2_ (RS), total CaO (TC), reactive calcium (RC), MgO, Al_2_O_3_, and Fe_2_O_3_; this compounds were also determined in a standard hydrochloric acid solution [18].

Pozzolanicity is the propensity of some substances to form a particular reaction when combined with Ca(OH)_2_ in a solution; when these materials have a very fine particle size, typically less than 50 microns [19], this property shines brightest. Pozzolanic materials can be made or grown naturally [20]. Using the guidelines of the Standard UNE-EN 196-5, this work examined two natural (IGNS-N) and calcined (IGNS-C) samples of ignimbrite at 8 and 15 days to determine their nature as pozzolans, using the guidelines of the Standard UNE-EN 196-5: 2011 [21]. For 60 s, 100 milliliters of distilled water were heated to 40 degrees Celsius; after that, 20 g each of PC/IGNS-N and PC/IGNS-C were added to this solution. The solutions were filtered after eight days. This test was retaken for a total of 15 days. The following equation was used to calculate the hydroxyl ion concentration (OH^−^):

(1) $$\left[{\mathrm{OH}}^{-}\right]=\frac{1000\times 0.1\times V_{3}\times f_{2}}{50}=2\times V_{3}\times f_{2}$$

where:

[OH^−^]: is the concentration in hydroxyl ions (mmol/L).
V_3_: is the volume of the hydrochloric acid solution (0.1 mol/L).
f_2_: is the factor of the hydrochloric acid solution (0.1 mol/L).

The concentration of calcium oxide (CaO) was calculated with the following equation:

(2) $$\left[\mathrm{CaO}\right]=\frac{1000\times 0.025\times V_{4}\times f_{1}}{50}=2\times V_{4}\times f_{1}$$

where:

[CaO]: is the concentration in calcium oxide (mmol/L).
V_4_: is the volume of the EDTA solution used in the titration.
f_1_: is the factor of the EDTA solution.

The pozzolanicity test is positive when the concentration of calcium hydroxide in solution is lower than the saturation concentration [21]. Thus, the results plotted on the corresponding graph will be below the solubility isotherm curve.

A mechanical resistance test was carried out to determine the compressive strength from specimens cured at 7, 28 and 90 days, using the guidelines of the Standard UNE-EN 196-1: 2018 [22]. The ignimbrite sample selected for this study was crushed three times. First, it was reduced in size to 3 cm in diameter with an “Alas” crusher (Madrid, Spain). Then, it was re-shredded to 1 cm in diameter with the “Controls” shredder. Two Blaine particle fractions (BPF) were then obtained with the aid of a “Siebtechnick” mill of the “Scheibenschwingmühle TS 1000” model: 2000 and 5000 cm^2^/g. For the finenesses 2000 and 5000 cm^2^/g the sample was ground for 50 and 30 s, respectively.

Standard mixtures of PC/IGNS-N and PC/IGNS-C were used in the making of the mortars for specimens. The percentage of PC substitution by IGNS-N and IGNS-C, respectively, was 10, 25 and 40%. In addition, two fine particle sizes (B.P.F.) of ignimbrite were included in the manufacture of the specimens, of 2000 and 5000 cm^2^/g, respectively. A total of 12 mortar specimens for the mechanical strength test were prepared, with PC/IGNS-N/C replacement percentages of 10, 25 and 40%. Table 1 provides all the data regarding the preparation and proportion of the components of the mortar specimens.

## 3. Results and Discussion

### 3.1. Study by Thin Section Petrographic (TSP)

**Figure 2:**
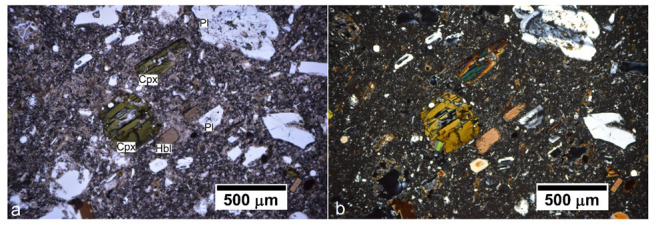
Transmitted light petrographic images under (**a**) plain-polarized, and (**b**) crossed-polarized light. Two big clinopyroxene (Cpx) phenocrystals, probably augite, abundant plagioclase (Pl), and some brown hornblende crystals surrounded by a sericitized groundmass are observed.

Under a petrographic microscope, the texture of the rock is porphyritic, containing abundant phenocrysts of zoned and twinned plagioclase and brown hornblende exhibiting yellow–brown pleochroism (Figure 2). Some biotite and clynopiroxene (augite) phenocrystals are also present. Quartz is rare and predominantly occurs as embayed phenocrysts ranging from 200 to 50 microns in size. Groundmass is composed mainly of small crystals of plagioclase and hardly distinguished augite almost fully altered. Abundant opaque minerals (probably magnetite or ilmenite) are observed in a devitrificated matrix completely sericitized.

### 3.2. X-ray Diffraction (XRD)

Mineralogical identification performed by XRD is concordant with chemical composition and petrographic observation since plagioclase, augite, quartz, hornblende, and magnetite have been recognized. K-feldspar diffraction peaks are also present then, most likely. This mineral is very small in size, is inside of the groundmass, and is incapable of being observed in thin section.

A broad band with two peaks at 15.0 and 14.5 Å, and another sharper one at 8.8 Å, corresponds to clays and phyllosilicates. For a perfect identification of clays and phyllosilicates minerals phases through enhancing their 00l reflections, XRD of oriented clay specimens was carried out, and smectite and illite were detected. Both minerals are secondary, formed by devitrification of the matrix and sericitization of feldspars.

### 3.3. Scanning Electron Microscopy (SEM)

**Figure 3:**
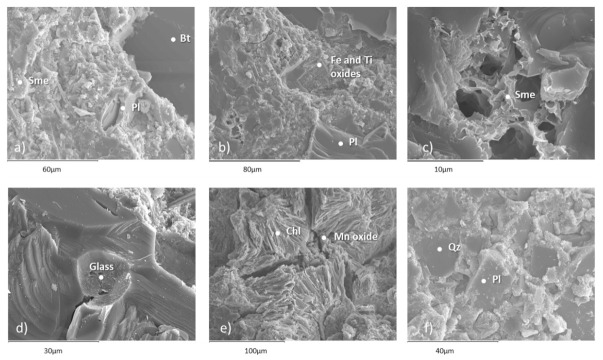
Microphotographs (**a**–**f**) obtained by scanning electron microscopy of secondary electrons. Pl: plagioclase; Bt: biotite; Sme: smectite; Chl: chlorite; Qz: quartz; Glass: volcanic glass.

**Figure 4:**
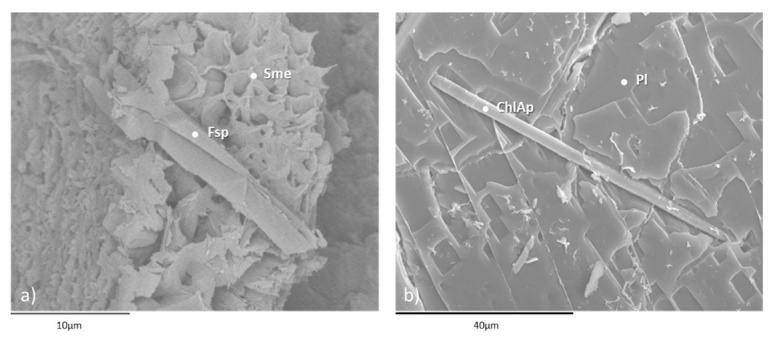
Microphotographs (**a**,**b**) obtained by scanning electron microscopy of backscattered electrons. Pl: plagioclase; Sme: smectite; ChlAp: chloroapatite; Fsp: feldspar.

SEM studies revealed the presence of mineral phases consisting of plagioclase, biotite, quartz, smectite, chlorite and volcanic glass (Figure 3). Backscattered electron analysis detected the presence of other phases, such as feldspar and chlorapatite (Figure 4), while confirming the mineral species mentioned in Figure 3.

### 3.4. Chemical Quality Analysis (QCA)

Chemical analysis performed by XRF shows essentially SiO_2_ and Al_2_O_3_ as the main components in 58.8%, and 16.4% by weight, respectively. Fe_2_O_3_, CaO, K_2_O, and MgO are also present in approximately 5% of each one, together with Na_2_O (2.0%) and TiO_2_ (0.6%).

Technical characterization tests using QCA to determine the quality of ignimbrite are shown in Table 2. Note how total SiO_2_ contents are remarkably high for natural ignimbrite samples (IGNS-N: 64.49%) and significantly higher for calcined ignimbrite (IGNS-C: 66.09%). This manifests differently for reactive SiO_2_ contents, where the values are higher for IGNS-N (20.28%) and somewhat lower for IGNS-C (19.28%). Total CaO presents a slight difference in both samples (IGNS-N: 4.32%; IGNS-C: 4.46%); a similar case occurs with Fe_2_O_3_ (3.59/3.69%) and Al_2_O_3_ (15.65%/15.87%).

The reactive CaO is increased in the IGNS-C sample (4.22%), which is equivalent to an increase of 0.4% in relation to the IGNS-N sample. The insoluble residue (IR) increases in the IGNS-C sample, which is equivalent to 5.12% more than that calculated for the IGNS-N sample. The values obtained from the SiO_2_/(CaO + MgO) ratio show a slight decrease when the sample passes from the natural phase to the calcined phase. Finally, the contents of SO_3_ and chlorides are highlighted. Table 2 shows the limit values allowed by the Standard UNE EN 196-2:2014 [18], referring to the calculated compounds. As can be seen, the requirements are met in practically all cases, except the insoluble residue (I.R.), which apparently portrays a negative aspect in this research caused by an excess of crystalline silica in the samples analyzed [23]. The results obtained highlight the fact that approximately 44% of the total SiO_2_ contained in the IGNS-N sample is able to react in the reaction system, whereas in the IGNS-C sample it is 47%. However, the SiO_2_ content in both samples is below the limit value indicated by the standard, although this fact does not affect in any way the pozzolanic and hydraulic reactivity of these samples, as discussed in Section 3.5 of this work. Costafreda [12] has already mentioned high values of pozzolanicity in their research with natural pozzolans enriched in SiO_2_. This aspect, together with the remarkable contents of CaO and MgO, is recurrent in the chalco-alkaline formations of southeastern Spain, which coincides with the conclusions of Costafreda et al. [24]. The behavior of compounds such as SiO_2_, Al_2_O_3_, reactive CaO, MgO and SO_3_ allows us to state that the samples studied can be considered as pozzolans, similar to what was established by Nurchasanah [25], Raggiottia et al. [26], Lim et al. [27] and Bellil et al. [28] in recent studies.

### 3.5. Pozzolanicity Test (PT)

**Figure 5:**
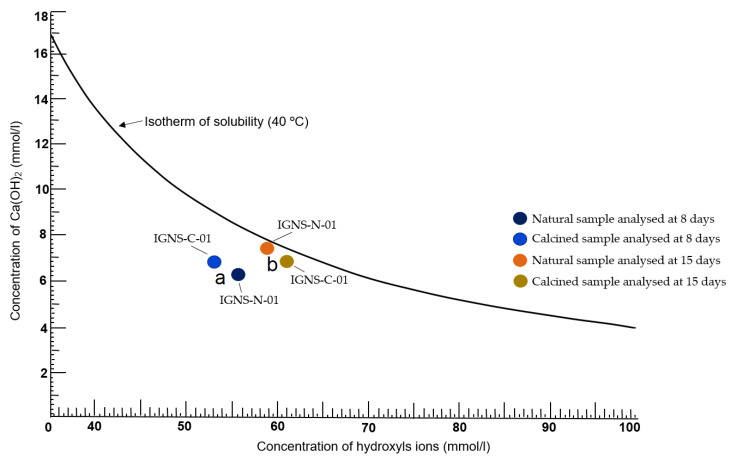
Pozzolanic behavior of ignimbrite samples analyzed at 8 (**a**) and 15 (**b**) days. The assay was conducted using natural (IGNS-N) and calcined (IGNS-C) ignimbrite samples.

Figure 5a,b show the results of the pozzolanicity test (PT) at 8 and 15 days. The position occupied by the samples under the isothermal curve of solubility at 40 °C indicates that the ignimbrite samples, in both the natural and calcined state, have pozzolanic behavior. An analysis of the results obtained allows us to establish that the pozzolanic character is manifested after 8 days of testing for both samples (Figure 5a). However, some differences are evident. For example, the IGNS-N sample shows higher pozzolanic activity than does the IGNS-C sample. This can be explained in the natural sample by the almost intact presence of clay, zeolitic and vitreous phases, which could favor pozzolanic reactivity. The work of Terci [29] shows that volcanic materials such as tuffs and ignimbrites can behave like very reactive pozzolans.

In the case of the calcined sample (IGNS-C), its low reactivity in relation to that of IGNS-N could be a consequence of the effect of heat on the physisorption phases in the sample, as well as the collapse of other phases and the deoxidization of smectite [12], where the new formed phases are less reactive.

After 15 days, a change in the position of both samples is observed (Figure 5b). The IGNS-C sample is now slightly more reactive than IGNS-N, as the latter completed its reaction with Ca(OH)_2_ earlier. Costafreda et al. [24] have shown that the change in position of the samples under the curve is proportional to the degree of reactivity of each sample. According to the results obtained and discussed, the objective of this work has been achieved in this phase of the research.

### 3.6. Mechanical Compressive Strength (MS) at 7, 28 and 90 Days

**Figure 6:**
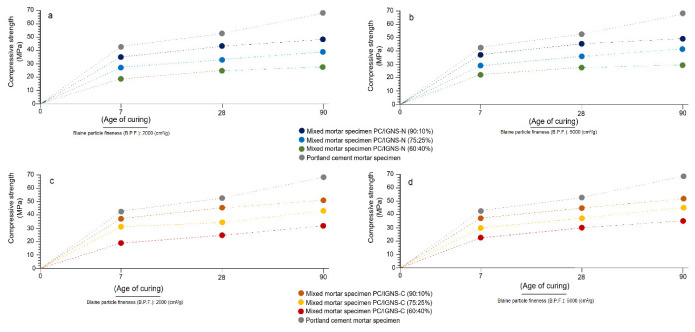
Mechanical compression strengths calculated from mortar specimens dosed with PC/IGNS and PCS at 7, 28 and 90 days. The (**a**,**b**) graphs represent the results obtained with the formulations of 10, 25 and 40% of natural IGNS and particle sizes of 2000 and 5000 cm^2^/g, respectively. A similar procedure is shown in (**c**,**d**), but with calcined samples.

Figure 6a–d show the results of the mechanical compressive strength test at 7, 28 and 90 days, which were obtained by testing mortar specimens with PC, PC/IGNS-N and PC/IGNS-C. The results show the variations of the mechanical strengths according to the degree of substitution of the PC by PC/IGNS-N and PC/IGNS-C (10, 25 and 40%), and according to the degree of fineness of the ignimbrite particles (B.P.F.), whether they were 2000 or 5000 cm^2^/g.

In the specific case of the 7-day mechanical strength results (B.P.F.: 2000 cm^2^/g) (Figure 6a), higher strength values are shown for the PC/IGNS-N formulations with 10% substitutions, followed by 25%. These values increase visibly when the B.P.F. is equal to 5000 cm^2^/g (Figure 6b).

As for the specimen made of PC/IGNS-C, an evident increase in mechanical strength is observed for the BPF:2000 and 5000 cm^2^/g finenesses at 7 days (Figure 6c,d). These values are comparatively higher than those calculated for the specimens made with PC/IGNS-N for the same curing period. This difference is due to the calcination process and to the increase in the specific surface area of the ignimbrite particles, which causes an improvement in their hydraulic and pozzolanic reactivity. Rosell-Lam et al. [30] have determined that the smaller the diameter of the pozzolan particles, the greater their tendency to increase their pozzolanic reactivity, and therefore increase mechanical strength.

After 28 days of curing, an increase in mechanical strength is still observed for both groups of specimens (PC/IGNS-C and PC/IGNS-C) (Figure 6a–d), with higher increases for the PC/IGNS-C formulations. The same trend is observed at 90 days of setting, where a discrete and gradual increase in mechanical strength persists for substitutions of 10, 25 and 40%. On the other hand, there is a directly proportional relationship among three aspects of this study: a lower percentage of PC/IGNS substitution, the degree of fineness of the particles (B.P.F.) and the mineral composition of the sample.

**Figure 7:**
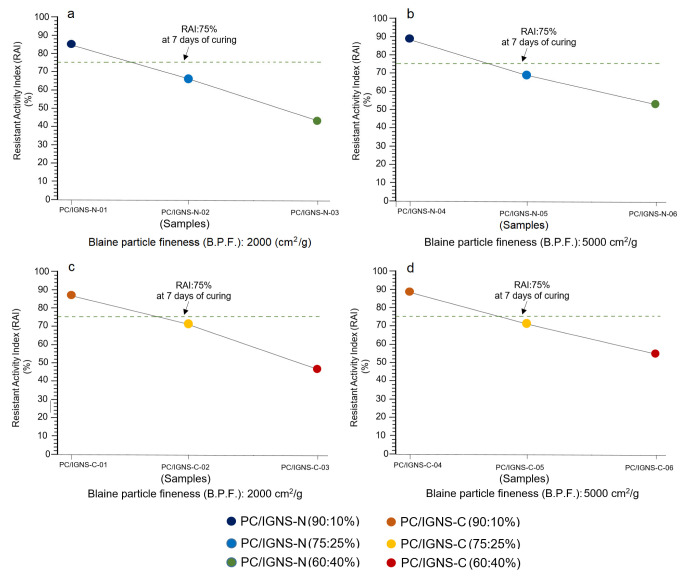
Evolution of the Resistant Activity Index (RAI) in mortar specimens made with mixtures of Portland cement (PC) and ignimbrite (PC/IGNS) in natural state (**a**,**b**) and calcined (**c**,**d**), and with fineness of IGNS particles of 2000 and 5000 cm^2^/g. RAI has been calculated for 7 days of curing.

Figure 7a–d show four graphs that re-create the variations of the Resistant Activity Index (RAI) at 7 days of setting. The RAI has been calculated based on the ratio of the mechanical strength value of the PC/IGNS-N and PC/IGNS-C mortar specimens and the PC specimens. In this ratio, the value of the mechanical strength of specimens made with ignimbrites must be greater than or equal to 75% of the mechanical strength of the reference mortar specimen (PC) [22]. This value is represented by a dotted line in Figure 7a–d.

The first aspect to highlight is that 75% of the RAI is exceeded when the replacement of the PC by natural and calcined ignimbrite is 10% for both particle sizes (2000 and 5000 cm^2^/g) (Figure 7a–d). Immediately below the line indicating the lower limit of the RAI are the samples with 25% substitution, and even further away are the samples with 40% substitution.

Some details are noteworthy in the specimens made with natural ignimbrite at 7 days of testing (Figure 7a,b). For example, for 10% substitutions and B.P.F.: 2000 cm^2^/g, the RAI is high (Figure 7a), but it is even higher for B.P.F.:5000 cm^2^/g (Figure 7b). In the replacement of 25% there is also a significant increase in RAI for B.P.F.: 5000 cm^2^/g.

The specimens made with calcined ignimbrites (PC/IGNS-C) at 7 days also show a trend similar to those described above, which are caused by the thermic treatment, by the degree of substitution of cement by ignimbrite and by the fineness of the ignimbrite particles, from 2000 to 5000 cm^2^/g (Figure 7c,d).

**Figure 8:**
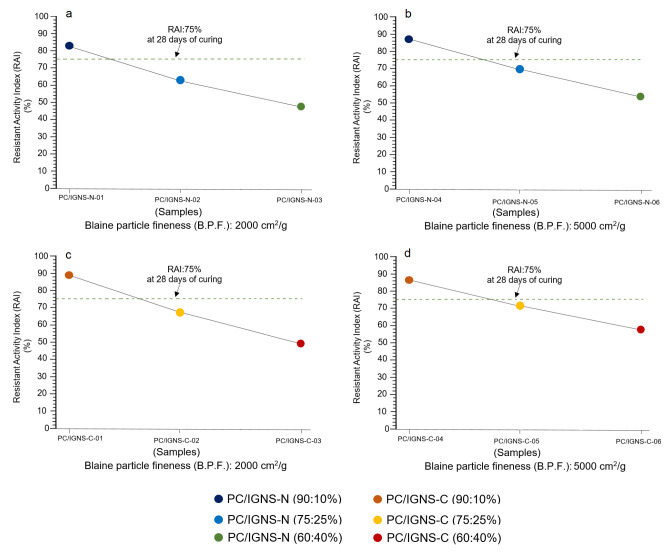
Variation of the Resistant Activity Index (RAI) in mortar specimens formulated with mixtures of Portland cement (PC) and ignimbrite (PC/IGNS) in natural state (**a**,**b**) and calcined (**c**,**d**), and with B.P.F. of the IGNS of 2000 and 5000 cm^2^/g, respectively. RAI was calculated for 28 days of curing.

The evolution of the Resistant Activity Index (RAI) calculated for 28 days of age shows an aspect relatively similar to that seen at 7 days (Figure 8a–d). It is observed that in all cases the specimens made with proportions of PC/IGNS: 10%, both natural and calcined, are above the line that indicates 75% of the RAI. Another aspect to consider is that in the samples of natural ignimbrite there is a significant difference that depends simultaneously on two factors: the degree of fineness [30] and the proportion of PC/IGNS [24]. In Figure 8a the position of the samples is further away from the limit of the RAI, in comparison to Figure 8b. A similar trend can be seen in Figure 8c,d, with the exception that after calcination the approximation of the values to the RAI line is more evident, due to the thermal process itself.

It can be construed that the transformations experienced by the samples during the grinding and calcination process significantly influence their pozzolanic and hydraulic properties, aspects that are adequately consolidated when compared with those established by Zhang and Ling [31], Zhou et al. [32], Florez et al. [33], Liu et al. [34] and Jaskulski et al. [35].

## 4. Conclusions

The analysis and discussion of the results obtained from the samples have led to the following conclusions:

1. The samples studied have pozzolanic behavior, according to the results obtained by the pozzolanicity test, thus fulfilling the fundamental objective of this work.
2. The samples have a mineral makeup that agrees with the mineralogy of the ignimbrites typical of the volcanic enclave of the Neogene, in the southeast of Spain, and are enriched with SiO_2_ and Al_2_O_3_ with a strong presence of chalco-alkaline compounds, which causes an evident tendency to pozzolanicity.
3. It is established that both the chemical and mineralogical compositions of the samples studied are decisive factors for the manifestation of their pozzolanic properties.
4. The specimens made with PC/IGNS-N and PC/IGNS-C substitutions of 10, 25 and 40% have mechanical strengths that increase from 7 to 90 days of curing.
5. The increase in compressive strengths becomes more evident as the degree of fineness (B.P.F.) increases from 2000 to 5000 cm^2^/g, and when the PC/IGNS ratio remains between 10 and 25%.
6. All specimens made with the PC:90%/IGNS:10% ratio have a Resistant Activity Index (RAI) above 75%, indicating that this formulation is the most suitable.
7. The degree of calcination of the sample also has a positive impact on the pozzolanic reactivity.
8. In order to increase profitability and quality in the production process, the parameters mentioned in the paragraph above should be taken into account, such as the degree of grinding, the degree of calcination and the dosing project of the PC/IGNS.

The results obtained in this research could be used in the manufacture of pozzolanic cements with low CO_2_ emissions to the atmosphere, as well as in the production of eco-efficient mortars and concretes.

## Figures and Tables

**Table 1:** Proportions of mixtures of PC/IGNS-N, PC/IGNS-C, NS and DW used in the preparation of mortar specimens.

Sample	Proportion (Ratios)				Temperature of Calcination (°C)	(B.P.F.) ^6^ (cm^2^/g)
	PC ^1^:IGNS-N ^2^ (%)	PC: IGNS-C ^3^ (%)	NS ^4^ (g)	DW ^5^ (g)		
IGNS-N-01	90:10	-	1.350	225	-	2000
IGNS-N-02	75:25					
IGNS-N-03	60:40					
IGNS-N-04	90:10					5000
IGNS-N-05	75:25					
IGNS-N-06	60:40					
IGNS-C-01	-	90:10	1.350	225	900	2000
IGNS-C-02		75:25				
IGNS-C-03		60:40				
IGNS-C-04		90:10				5000
IGNS-C-05		75:25				
IGNS-C-06		60:40				

^1^ Portland cement; ^2^ natural ignimbrite; ^3^ calcined ignimbrite; ^4^ normalized sand; ^5^ distilled water; ^6^ Blaine particle fineness.

**Table 2:** Results of the chemical analysis of quality of the samples.

% Weight	Samples		Allowed Levels (%)
	IGNS-N	IGNS-C	
Total SiO_2_	64.49	66.09	-
MgO	2.22	2.29	<5
Total CaO	4.32	4.46	-
Fe_2_O_3_	3.59	3.69	-
Al_2_O_3_	15.65	15.87	<16
Reactive SiO_2_	20.28	19.28	>25
Reactive CaO	3.82	4.22	-
Insoluble Residue	61.97	67.09	<3
Loss on Ignition (LOI)	2.76	0.43	-
SiO_2_/(CaO + MgO)	10.70	10.20	>3.5
SO_3_	0.038	0.013	<4
Chlorides	0.021	0.008	<0.1

## Data Availability

Not applicable.
